# Supplementary material for: Antibiotic Prescription in the Community-Dwelling Elderly Population in Lombardy, Italy: A Sub-Analysis of the EDU.RE.DRUG Study
Source: Antibiotics (Basel). 2022 Oct 7;11(10):1369. doi: 10.3390/antibiotics11101369 (PMC9599035; doi:10.3390/antibiotics11101369)
Supplement: Supplementary file 1 [file antibiotics-11-01369-s001.zip › antibiotics-1944480-supplementary.pdf]

SUPPLEMENTARY MATERIAL

Antibiotic Prescription in the Community-Dwelling Elderly Population in Lombardy, Italy: A Sub-Analysis of the EDU.RE.DRUG Study

Table S1 – Characteristics of the resident citizens (≥65 years) and their general practitioners (GP) in Lombardy region and by local health unit (LHU)

	**Lombardy***	**Bergamo**	**Lecco**	**Mantova**	**Monza Brianza**	**4 LHUs combined**
			**CITIZENS (N)**			
**≥65 ys**	**2269109**	**234053**	**79512**	**97529**	**195766**	**606860**
65-74 ys	1095770	118883	39170	46446	95014	299513
75-84 ys	826847	82429	28366	34907	72031	217733
≥85 ys	346492	32741	11976	16176	28721	89614
			**MALES (N)**			
**≥65 ys**	**977542**	**103521**	**35043**	**41819**	**85640**	**266023**
65-74 ys	516603	57620	18882	22020	44755	143277
75-84 ys	354322	35869	12435	14943	31566	94813
≥85 ys	106617	10032	3726	4856	9319	27933
			**FEMALES (N)**			
**≥65 ys**	**1291567**	**130532**	**44469**	**55710**	**110126**	**340837**
65-74 ys	579167	61263	20288	24426	50259	156236
75-84 ys	472525	46560	15931	19964	40465	122920
≥85 ys	239875	22709	8250	11320	19402	61681
			**GP (N)**			
**Overall**	**6218**	**644**	**214**	**266**	**519**	**1643**
Males	3924	414	147	164	314	1039
Females	2294	230	67	102	205	604

*data retrieved from ISTAT (https://demo.istat.it/popres/index.php?anno=2019&lingua=ita) and Ministry of Health websites (https://www.salute.gov.it/portale/documentazione/p6_2_2_1.jsp?lingua=italiano&id=2980)

Table S2 - ESAC-based indicators

**ESAC 1**	J01_DID	Consumption of antibacterials for systemic use (J01) expressed in DID
**ESAC 2**	J01C_DID	Consumption of penicillins (J01C) expressed in DID
**ESAC 3**	J01D_DID	Consumption of cephalosporins (J01D) expressed in DID
**ESAC 4**	J01F_DID	Consumption of macrolides, lincosamides and streptogramins (J01F) expressed in DID
**ESAC 5**	J01M_DID	Consumption of quinolones (J01M) expressed in DID
**ESAC 6**	J01CE_%	Consumption of beta-lactamase sensitive penicillins (J01CE) expressed as percentage of the total consumption of antibacterials for systemic use (J01)
**ESAC 7**	J01CR_%	Consumption of combination of penicillins, including beta-lactamase inhibitor (J01CR) expressed as percentage of the total consumption of antibacterials for systemic use (J01)
**ESAC 8**	J01DD+DE_%	Consumption of third- and fourth-generation cephalosporins (J01(DD+DE)) expressed as percentage of the total consumption of antibacterials for systemic use (J01)
**ESAC 9**	J01MA_%	Consumption of fluoroquinolones (J01MA) expressed as percentage of the total consumption of antibacterials for systemic use (J01)
**ESAC 10**	J01_B/N	Ratio of consumption of broad-spectrum penicillins, cephalosporins, macrolides (except erythromycin) and fluoroquinolones (J01(CR+DC+DD+(FA–FA01)+MA)) to the consumption of narrow-spectrum penicillins, cephalosporins and erythromycin (J01(CA+CE+CF+DB+FA01))
**ESAC 11**	J01_SV	Seasonal variation (SV) of the total antibiotic consumption (J01)<br>SV= [Winter (January, February, March, October, November, and December) consumption expressed in DID/ Summer (April, May, June, July, August and September) consumption expressed in DID)-1] x100
**ESAC 12**	J01M_SV	Seasonal variation of quinolone consumption (J01M)<br>SV= [Winter (January, February, March, October, November, and December) consumption expressed in DID/ Summer (April, May, June, July, August and September) consumption expressed in DID)-1] x100

Table S3 - WHO-AWaRe classification, 2021

Antibiotic	Class	ATC code	Category
Amikacin	Aminoglycosides	J01GB06	Access
Amoxicillin	Penicillins	J01CA04	Access
Amoxicillin/clavulanic-acid	Beta-lactam/beta-lactamase-inhibitor	J01CR02	Access
Ampicillin	Penicillins	J01CA01	Access
Ampicillin/sulbactam	Beta-lactam/beta-lactamase-inhibitor	J01CR01	Access
Arbekacin	Aminoglycosides	J01GB12	Watch
Aspoxicillin	Penicillins	J01CA19	Watch
Azidocillin	Penicillins	J01CE04	Access
Azithromycin	Macrolides	J01FA10	Watch
Azlocillin	Penicillins	J01CA09	Watch
Aztreonam	Monobactams	J01DF01	Reserve
Bacampicillin	Penicillins	J01CA06	Access
Bekanamycin	Aminoglycosides	J01GB13	Watch
Benzathine-benzylpenicillin	Penicillins	J01CE08	Access
Benzylpenicillin	Penicillins	J01CE01	Access
Biapenem	Carbapenems	J01DH05	Watch
Brodimoprim	Trimethoprim-derivatives	J01EA02	Access
Carbenicillin	Penicillins	J01CA03	Watch
Carindacillin	Penicillins	J01CA05	Watch
Carumonam	Monobactams	J01DF02	Reserve
Cefacetrile	First-generation-cephalosporins	J01DB10	Access
Cefaclor	Second-generation-cephalosporins	J01DC04	Watch
Cefadroxil	First-generation-cephalosporins	J01DB05	Access
Cefalexin	First-generation-cephalosporins	J01DB01	Access
Cefaloridine	First-generation-cephalosporins	J01DB02	Access
Cefalotin	First-generation-cephalosporins	J01DB03	Access
Cefamandole	Second-generation-cephalosporins	J01DC03	Watch
Cefapirin	First-generation-cephalosporins	J01DB08	Access
Cefatrizine	First-generation-cephalosporins	J01DB07	Access
Cefazedone	First-generation-cephalosporins	J01DB06	Access
Cefazolin	First-generation-cephalosporins	J01DB04	Access
Cefbuperazone	Second-generation-cephalosporins	J01DC13	Watch
Cefcapene-pivoxil	Third-generation-cephalosporins	J01DD17	Watch
Cefdinir	Third-generation-cephalosporins	J01DD15	Watch
Cefditoren-pivoxil	Third-generation-cephalosporins	J01DD16	Watch
Cefepime	Fourth-generation-cephalosporins	J01DE01	Watch
Cefetamet-pivoxil	Third-generation-cephalosporins	J01DD10	Watch
Cefiderocol	Other-cephalosporins	J01DI04	Reserve
Cefixime	Third-generation-cephalosporins	J01DD08	Watch
Cefmenoxime	Third-generation-cephalosporins	J01DD05	Watch
Cefmetazole	Second-generation-cephalosporins	J01DC09	Watch
Cefminox	Second-generation-cephalosporins	J01DC12	Watch
Cefodizime	Third-generation-cephalosporins	J01DD09	Watch
Cefonicid	Second-generation-cephalosporins	J01DC06	Watch
Cefoperazone	Third-generation-cephalosporins	J01DD12	Watch
Ceforanide	Second-generation-cephalosporins	J01DC11	Watch

Cefoselis	Fourth-generation-cephalosporins	to be assigned	Watch
Cefotaxime	Third-generation-cephalosporins	J01DD01	Watch
Cefotetan	Second-generation-cephalosporins	J01DC05	Watch
Cefotiam	Second-generation-cephalosporins	J01DC07	Watch
Cefoxitin	Second-generation-cephalosporins	J01DC01	Watch
Cefozopran	Fourth-generation-cephalosporins	J01DE03	Watch
Cefpiramide	Third-generation-cephalosporins	J01DD11	Watch
Cefpirome	Fourth-generation-cephalosporins	J01DE02	Watch
Cefpodoxime-proxetil	Third-generation-cephalosporins	J01DD13	Watch
Cefprozil	Second-generation-cephalosporins	J01DC10	Watch
Cefradine	First-generation-cephalosporins	J01DB09	Access
Cefroxadine	First-generation-cephalosporins	J01DB11	Access
Cefsulodin	Third-generation-cephalosporins	J01DD03	Watch
Ceftaroline-fosamil	Fifth-generation cephalosporins	J01DI02	Reserve
Ceftazidime	Third-generation-cephalosporins	J01DD02	Watch
Ceftazidime/avibactam	Third-generation-cephalosporins	J01DD52	Reserve
Cefteram-pivoxil	Third-generation-cephalosporins	J01DD18	Watch
Ceftezole	First-generation-cephalosporins	J01DB12	Access
Ceftibuten	Third-generation-cephalosporins	J01DD14	Watch
Ceftizoxime	Third-generation-cephalosporins	J01DD07	Watch
Ceftobiprole-medocaril	Fifth-generation cephalosporins	J01DI01	Reserve
Ceftolozane/tazobactam	Fifth-generation cephalosporins	J01DI54	Reserve
Ceftriaxone	Third-generation-cephalosporins	J01DD04	Watch
Cefuroxime	Second-generation-cephalosporins	J01DC02	Watch
Chloramphenicol	Amphenicols	J01BA01	Access
Chlortetracycline	Tetracyclines	J01AA03	Watch
Cinoxacin	Quinolones	J01MB06	Watch
Ciprofloxacin	Fluoroquinolones	J01MA02	Watch
Clarithromycin	Macrolides	J01FA09	Watch
Clindamycin	Lincosamides	J01FF01	Access
Clofoctol	Phenol derivatives	J01XX03	Watch
Clometocillin	Penicillins	J01CE07	Access
Clomocycline	Tetracyclines	J01AA11	Watch
Cloxacillin	Penicillins	J01CF02	Access
Colistin_IV	Polymyxins	J01XB01	Reserve
Colistin_oral	Polymyxins	A07AA10	Reserve
Dalbavancin	Glycopeptides	J01XA04	Reserve
Dalfopristin/quinupristin	Streptogramins	J01FG02	Reserve
Daptomycin	Lipopeptides	J01XX09	Reserve
Delafloxacin	Fluoroquinolones	J01MA23	Watch
Demeclocycline	Tetracyclines	J01AA01	Watch
Dibekacin	Aminoglycosides	J01GB09	Watch
Dicloxacillin	Penicillins	J01CF01	Access
Dirithromycin	Macrolides	J01FA13	Watch
Doripenem	Carbapenems	J01DH04	Watch
Doxycycline	Tetracyclines	J01AA02	Access
Enoxacin	Fluoroquinolones	J01MA04	Watch
Epicillin	Penicillins	J01CA07	Access

Eravacycline	Tetracyclines	J01AA13	Reserve
Ertapenem	Carbapenems	J01DH03	Watch
Erythromycin	Macrolides	J01FA01	Watch
Faropenem	Penems	J01DI03	Reserve
Fidaxomicin	Macrolides	A07AA12	Watch
Fleroxacin	Fluoroquinolones	J01MA08	Watch
Flomoxef	Second-generation-cephalosporins	J01DC14	Watch
Flucloxacillin	Penicillins	J01CF05	Access
Flumequine	Quinolones	J01MB07	Watch
Flurithromycin	Macrolides	J01FA14	Watch
Fosfomycin_IV	Phosphonics	J01XX01	Reserve
Fosfomycin_oral	Phosphonics	J01XX01	Watch
Furazidin	Nitrofuran derivatives	J01XE03	Access
Fusidic-acid	Steroid antibacterials	J01XC01	Watch
Garenoxacin	Fluoroquinolones	J01MA19	Watch
Gatifloxacin	Fluoroquinolones	J01MA16	Watch
Gemifloxacin	Fluoroquinolones	J01MA15	Watch
Gentamicin	Aminoglycosides	J01GB03	Access
Grepafloxacin	Fluoroquinolones	J01MA11	Watch
Hetacillin	Penicillins	J01CA18	Access
Iclaprim	Trimethoprim-derivatives	J01EA03	Reserve
Imipenem/cilastatin	Carbapenems	J01DH51	Watch
Imipenem/cilastatin/relebactam	Carbapenems	J01DH56	Reserve
Isepamicin	Aminoglycosides	J01GB11	Watch
Josamycin	Macrolides	J01FA07	Watch
Kanamycin_IV	Aminoglycosides	J01GB04	Watch
Kanamycin_oral	Aminoglycosides	A07AA08	Watch
Lascufloxacin	Fluoroquinolones	J01MA25	Watch
Latamoxef	Third-generation-cephalosporins	J01DD06	Watch
Lefamulin	Pleuromutilin	J01XX12	Reserve
Levofloxacin	Fluoroquinolones	J01MA12	Watch
Levonadifloxacin	Fluoroquinolones	J01MA24	Watch
Lincomycin	Lincosamides	J01FF02	Watch
Linezolid	Oxazolidinones	J01XX08	Reserve
Lomefloxacin	Fluoroquinolones	J01MA07	Watch
Loracarbef	Second-generation-cephalosporins	J01DC08	Watch
Lymecycline	Tetracyclines	J01AA04	Watch
Mecillinam	Penicillins	J01CA11	Access
Meropenem	Carbapenems	J01DH02	Watch
Meropenem/vaborbactam	Carbapenems	J01DH52	Reserve
Metacycline	Tetracyclines	J01AA05	Watch
Metampicillin	Penicillins	J01CA14	Access
Meticillin	Penicillins	J01CF03	Access
Metronidazole_IV	Imidazoles	J01XD01	Access
Metronidazole_oral	Imidazoles	P01AB01	Access
Mezlocillin	Penicillins	J01CA10	Watch
Micronomicin	Aminoglycosides	to be assigned	Watch
Midecamycin	Macrolides	J01FA03	Watch

Minocycline_IV	Tetracyclines	J01AA08	Reserve
Minocycline_oral	Tetracyclines	J01AA08	Watch
Miocamycin	Macrolides	J01FA11	Watch
Moxifloxacin	Fluoroquinolones	J01MA14	Watch
Nafcillin	Penicillins	J01CF06	Access
Nemonoxacin	Quinolones	J01MB08	Watch
Neomycin_IV	Aminoglycosides	J01GB05	Watch
Neomycin_oral	Aminoglycosides	A07AA01	Watch
Netilmicin	Aminoglycosides	J01GB07	Watch
Nifurtoinol	Nitrofuran derivatives	J01XE02	Access
Nitrofurantoin	Nitrofuran-derivatives	J01XE01	Access
Norfloxacin	Fluoroquinolones	J01MA06	Watch
Ofloxacin	Fluoroquinolones	J01MA01	Watch
Oleandomycin	Macrolides	J01FA05	Watch
Omadacycline	Tetracyclines	J01AA15	Reserve
Oritavancin	Glycopeptides	J01XA05	Reserve
Ornidazole_IV	Imidazoles	J01XD03	Access
Ornidazole_oral	Imidazoles	P01AB03	Access
Oxacillin	Penicillins	J01CF04	Access
Oxolinic-acid	Quinolones	J01MB05	Watch
Oxytetracycline	Tetracyclines	J01AA06	Watch
Panipenem	Carbapenems	J01DH55	Watch
Pazufloxacin	Fluoroquinolones	J01MA18	Watch
Pefloxacin	Fluoroquinolones	J01MA03	Watch
Penamecillin	Penicillins	J01CE06	Access
Penimepicycline	Tetracyclines	J01AA10	Watch
Pheneticillin	Penicillins	J01CE05	Watch
Phenoxymethylpenicillin	Penicillins	J01CE02	Access
Pipemidic-acid	Quinolones	J01MB04	Watch
Piperacillin	Penicillins	J01CA12	Watch
Piperacillin/tazobactam	Beta-lactam/beta-lactamase-inhibitor_anti-pseudomonal	J01CR05	Watch
Piromidic-acid	Quinolones	J01MB03	Watch
Pivampicillin	Penicillins	J01CA02	Access
Pivmecillinam	Penicillins	J01CA08	Access
Plazomicin	Aminoglycosides	J01GB14	Reserve
Polymyxin-B_IV	Polymyxins	J01XB02	Reserve
Polymyxin-B_oral	Polymyxins	A07AA05	Reserve
Pristinamycin	Streptogramins	J01FG01	Watch
Procaine-benzylpenicillin	Penicillins	J01CE09	Access
Propicillin	Penicillins	J01CE03	Access
Prulifloxacin	Fluoroquinolones	J01MA17	Watch
Ribostamycin	Aminoglycosides	J01GB10	Watch
Rifabutin	Rifamycins	J04AB04	Watch
Rifampicin	Rifamycins	J04AB02	Watch
Rifamycin_IV	Rifamycins	J04AB03	Watch
Rifamycin_oral	Rifamycins	A07AA13	Watch
Rifaximin	Rifamycins	A07AA11	Watch
Rokitamycin	Macrolides	J01FA12	Watch

Rolitetracycline	Tetracyclines	J01AA09	Watch
Rosoxacin	Quinolones	J01MB01	Watch
Roxithromycin	Macrolides	J01FA06	Watch
Rufloxacin	Fluoroquinolones	J01MA10	Watch
Sarecycline	Tetracyclines	J01AA14	Watch
Secnidazole	Imidazoles	P01AB07	Access
Sisomicin	Aminoglycosides	J01GB08	Watch
Sitafloxacin	Fluoroquinolones	J01MA21	Watch
Solithromycin	Macrolides	J01FA16	Watch
Sparfloxacin	Fluoroquinolones	J01MA09	Watch
Spectinomycin	Aminocyclitols	J01XX04	Access
Spiramycin	Macrolides	J01FA02	Watch
Spiramycin/metronidazole	Antibacterials_combinations	J01RA04	Watch
Streptoduocin	Aminoglycosides	J01GA02	Watch
Streptomycin_IV	Aminoglycosides	J01GA01	Watch
Streptomycin_oral	Aminoglycosides	A07AA04	Watch
Sulbactam	Beta-lactamase-inhibitors	J01CG01	Access
Sulbenicillin	Penicillins	J01CA16	Watch
Sulfadiazine	Sulfonamides	J01EC02	Access
Sulfadiazine/tetroxoprim	Sulfonamide-trimethoprim-combinations	J01EE06	Access
Sulfadiazine/trimethoprim	Sulfonamide-trimethoprim-combinations	J01EE02	Access
Sulfadimethoxine	Sulfonamides	J01ED01	Access
Sulfadimidine	Sulfonamides	J01EB03	Access
Sulfadimidine/trimethoprim	Sulfonamide-trimethoprim-combinations	J01EE05	Access
Sulfafurazole	Sulfonamides	J01EB05	Access
Sulfaisodimidine	Sulfonamides	J01EB01	Access
Sulfalene	Sulfonamides	J01ED02	Access
Sulfamazone	Sulfonamides	J01ED09	Access
Sulfamerazine	Sulfonamides	J01ED07	Access
Sulfamerazine/trimethoprim	Sulfonamide-trimethoprim-combinations	J01EE07	Access
Sulfamethizole	Sulfonamides	J01EB02	Access
Sulfamethoxazole	Sulfonamides	J01EC01	Access
Sulfamethoxazole/trimethoprim	Sulfonamide-trimethoprim-combinations	J01EE01	Access
Sulfamethoxypyridazine	Sulfonamides	J01ED05	Access
Sulfametomidine	Sulfonamides	J01ED03	Access
Sulfametoxydiazine	Sulfonamides	J01ED04	Access
Sulfametrole/trimethoprim	Sulfonamide-trimethoprim-combinations	J01EE03	Access
Sulfamoxole	Sulfonamides	J01EC03	Access
Sulfamoxole/trimethoprim	Sulfonamide-trimethoprim-combinations	J01EE04	Access
Sulfanilamide	Sulfonamides	J01EB06	Access
Sulfaperin	Sulfonamides	J01ED06	Access
Sulfaphenazole	Sulfonamides	J01ED08	Access
Sulfapyridine	Sulfonamides	J01EB04	Access
Sulfathiazole	Sulfonamides	J01EB07	Access
Sulfathiourea	Sulfonamides	J01EB08	Access
Sultamicillin	Beta-lactam/beta-lactamase-inhibitor	J01CR04	Access
Talampicillin	Penicillins	J01CA15	Access
Tazobactam	Beta-lactamase-inhibitors	J01CG02	Watch
Tebipenem	Carbapenems	J01DH06	Watch

Tedizolid	Oxazolidinones	J01XX11	Reserve
Teicoplanin	Glycopeptides	J01XA02	Watch
Telavancin	Glycopeptides	J01XA03	Reserve
Telithromycin	Macrolides	J01FA15	Watch
Temafloxacin	Fluoroquinolones	J01MA05	Watch
Temocillin	Penicillins	J01CA17	Watch
Tetracycline	Tetracyclines	J01AA07	Access
Thiamphenicol	Amphenicols	J01BA02	Access
Ticarcillin	Penicillins	J01CA13	Watch
Tigecycline	Glycylcyclines	J01AA12	Reserve
Tinidazole_IV	Imidazoles	J01XD02	Access
Tinidazole_oral	Imidazoles	P01AB02	Access
Tobramycin	Aminoglycosides	J01GB01	Watch
Tosufloxacin	Fluoroquinolones	J01MA22	Watch
Trimethoprim	Trimethoprim-derivatives	J01EA01	Access
Troleandomycin	Macrolides	J01FA08	Watch
Trovafloxacin	Fluoroquinolones	J01MA13	Watch
Vancomycin_IV	Glycopeptides	J01XA01	Watch
Vancomycin_oral	Glycopeptides	A07AA09	Watch

Table S4 - Antibiotic consumption per patient, stratified by sex and age groups

	Overall	Males	Females
**J01 DDD/patient**			
65-74 ys	15.95	16.87	15.20
75-84 ys	16.02	17.42	14.95
≥85 ys	16.68	18.05	15.99
**J01 PACKAGES/patient**			
65-74 ys	3.15	3.28	3.05
75-84 ys	3.53	3.76	3.36
≥85 ys	4.43	4.60	4.35
**J01 POSOLOGIC UNITS/patient**			
65-74 ys	21.62	22.82	20.63
75-84 ys	21.80	23.55	20.47
≥85 ys	22.80	24.37	22.01

Table S5 - DID consumption for each antibiotic class (ATC 3rd level) classified according to the WHO-AWaRe list (Access and Watch) and stratified by sex and age groups

Antibiotic classes (ATC 3rd level)	Males		Females	
	Access	Watch	Access	Watch
**65-74 years**				
Aminoglycosides	0.0088	0.0032	0.0050	0.0012
Combinations of sulfonamides and trimethoprim	0.3501		0.2210	
First-generation cephalosporins	0.1373		0.0612	
Fluoroquinolones		4.3122		3.4842
Fourth-generation cephalosporins		0.0031		0.0029
Glycopeptides		0.0066		0.0018
Imidazoles			0.00002	
Lincosamides	0.1373		0.0052	
Macrolides		2.6369		3.5810
Penicillins	1.0570	0.0005	1.2819	0.0001
Penicillins, combinations with beta lactamase inhibitors	5.0255		5.0346	
Penicillins, combinations with beta lactamase inhibitors (anti-pseudomonal)		0.0114		0.0011
Phosphonics		1.0472		0.6021
Second-generation cephalosporins		0.0808		0.0936
Tetracyclines	0.2329	0.0182	0.1582	0.0331
Third-generation cephalosporins		2.6485		1.5179
**75-84 years**				
Aminoglycosides	0.0105	0.0017	0.0095	0.0023
Combinations of sulfonamides and trimethoprim	0.3927		0.2481	
First-generation cephalosporins	0.1249		0.1017	
Fluoroquinolones		5.4886		3.9376
Fourth-generation cephalosporins		0.0066		0.0046
Glycopeptides		0.0027		0.0024
Imidazoles	0.0001		0.0002	
Lincosamides	0.0033		0.0022	
Macrolides		2.8671		2.9402
Penicillins	0.9971	0.0003	1.0675	0.0004
Penicillins, combinations with beta lactamase inhibitors	5.5040		4.6896	
Penicillins, combinations with beta lactamase inhibitors (anti-pseudomonal)		0.0035		0.0024
Phosphonics		0.3395		0.9089
Second-generation cephalosporins		0.0989		0.0850
Tetracyclines	0.2433	0.0737	0.1253	0.0267
Third-generation cephalosporins		1.9597		1.7604
**≥85 years**				
Aminoglycosides	0.0332	0.0045	0.0149	0.0026

Combinations of sulfonamides and trimethoprim	0.3271		0.2551	
First-generation cephalosporins	0.1294		0.1159	
Fluoroquinolones		6.6340		4.1972
Fourth-generation cephalosporins		0.0097		0.0072
Glycopeptides		0.0109		0.0036
Imidazoles			0.0003	
Lincosamides	0.0043		0.0028	
Macrolides		2.9891		2.4774
Penicillins	0.8290	0.00001	0.7853	0.0007
Penicillins, combinations with beta lactamase inhibitors	6.0317		4.8716	
Penicillins, combinations with beta lactamase inhibitors (anti-pseudomonal)		0.0097		0.0122
Phosphonics		0.7859		1.1655
Second-generation cephalosporins		0.0763		0.0828
Tetracyclines	0.2942	0.0261	0.2061	0.0146
Third-generation cephalosporins		2.9420		2.5156

Table S6 – Rank of antibiotic substances (ATC 5th level) based on consumption in DID, classified according to the WHO-AWaRe list (Access and Watch) and stratified by sex (**panel a**: Overall; **panel b**: Males; **panel c**: Females)

(a) Overall

Rank	Antibiotic name	ATC 5th level	DDD N	DDD %	DID	AWaRe Class
1	*amoxicillin+clavulanic acid*	J01CR02	1121952.69	30.54%	5.07	Access
2	*levofloxacin*	J01MA12	610010.00	16.60%	2.75	Watch
3	*clarithromicyn*	J01FA09	341759.00	9.30%	1.54	Watch
4	*azithromicyn*	J01FA10	314325.00	8.56%	1.42	Watch
5	*ciprofloxacin*	J01MA02	266686.50	7.26%	1.20	Watch
6	*amoxicillin*	J01CA04	231910.26	6.31%	1.05	Access
7	*cefixime*	J01DD08	227715.00	6.20%	1.03	Watch
8	*fosfomycin*	J01XX01	129602.67	3.53%	0.59	Watch
9	*trimetoprim+sulfamethoxazole*	J01EE01	64713.00	1.76%	0.29	Access
10	*ceftriaxone*	J01DD04	64177.50	1.75%	0.29	Watch
11	*cefditoren*	J01DD16	57460.00	1.56%	0.26	Watch
12	*doxycycline*	J01AA02	42880.00	1.17%	0.19	Access
13	*prulifloxacin*	J01MA17	41158.00	1.12%	0.19	Watch
14	*ceftibuten*	J01DD14	24991.20	0.68%	0.11	Watch
15	*cefalexina*	J01DB01	23386.50	0.64%	0.11	Access
16	*norfloxacin*	J01MA06	18067.00	0.49%	0.08	Watch
17	*cefuroxima*	J01DC02	16254.00	0.44%	0.07	Watch
18	*cefpodoxime*	J01DD13	14869.00	0.40%	0.07	Watch
19	*moxifloxacin*	J01MA14	7810.00	0.21%	0.04	Watch
20	*spiramycin*	J01FA02	7704.00	0.21%	0.03	Watch
21	*minocycline*	J01AA08	7508.00	0.20%	0.03	Watch
22	*roxithromycin*	J01FA06	6198.00	0.17%	0.03	Watch
23	*lomefloxacin*	J01MA07	5415.00	0.15%	0.02	Watch
24	*bacampicillin*	J01CA06	4332.00	0.12%	0.02	Access
25	*pipemidic acid*	J01MB04	3090.00	0.08%	0.01	Watch
26	*cefaclor*	J01DC04	3014.50	0.08%	0.01	Watch
27	*lymecycline*	J01AA04	2688.00	0.07%	0.01	Watch
28	*amikacin*	J01GB06	2206.00	0.06%	0.01	Access
29	*ceftazidime*	J01DD02	1849.38	0.05%	0.01	Watch
30	*miocamycin*	J01FA11	1848.00	0.05%	0.01	Watch
31	*cefodizime*	J01DD09	1196.50	0.03%	0.01	Watch
32	*cefepime*	J01DE01	1027.50	0.03%	<0.01	Watch
33	*teicoplanin*	J01XA02	839.50	0.02%	<0.01	Watch
34	*clindamycin*	J01FF01	794.33	0.02%	<0.01	Access
35	*piperacillin+tazobactam*	J01CR05	770.00	0.02%	<0.01	Watch
36	*cefotaxime*	J01DD01	708.00	0.02%	<0.01	Watch
37	*flucloxacillin*	J01CF05	618.00	0.02%	<0.01	Access
38	*pefloxacin*	J01MA03	572.00	0.02%	<0.01	Watch

39	*lincomycin*	J01FF02	555.33	0.02%	<0.01	Watch
40	*cefprozil*	J01DC10	285.00	0.01%	<0.01	Watch
41	*netilmicin*	J01GB07	259.29	0.01%	<0.01	Watch
42	*tobramycin*	J01GB01	247.71	0.01%	<0.01	Watch
43	*josamycin*	J01FA07	171.00	<0.01%	<0.01	Watch
44	*rufloxacin*	J01MA10	132.00	<0.01%	<0.01	Watch
45	*cefazolin*	J01DB04	98.00	<0.01%	<0.01	Access
46	*telithromycin*	J01FA15	70.00	<0.01%	<0.01	Watch
47	*piperacillin*	J01CA12	53.00	<0.01%	<0.01	Watch
48	*erythromycin*	J01FA01	42.00	<0.01%	<0.01	Watch
49	*benzathine benzylpenicillin*	J01CE08	36.25	<0.01%	<0.01	Access
50	*metronidazole*	J01XD01	19.00	<0.01%	<0.01	Access
51	*ampicillin+sulbactam*	J01CR01	17.17	<0.01%	<0.01	Access
52	*oxacillin*	J01CF04	16.50	<0.01%	<0.01	Access
53	*cefoxitin*	J01DC01	3.50	<0.01%	<0.01	Watch

(b) Males

Rank	Antibiotic name	ATC 5th level	DDD		DID	AWaRe Class
			N	%		
1	*amoxicillin+clavulanic acid*	J01CR02	514776.70	30.90%	5.3	Access
2	*levofloxacin*	J01MA12	324385.00	19.47%	3.34	Watch
3	*clarithromicyn*	J01FA09	141265.00	8.48%	1.45	Watch
4	*azithromicyn*	J01FA10	133132.00	7.99%	1.37	Watch
5	*ciprofloxacin*	J01MA02	124146.50	7.45%	1.28	Watch
6	*amoxicillin*	J01CA04	95972.65	5.76%	0.99	Access
7	*cefixime*	J01DD08	95650.00	5.74%	0.99	Watch
8	*trimetoprim+sulfamethoxazole*	J01EE01	35234.00	2.11%	0.36	Access
9	*ceftriaxone*	J01DD04	28992.00	1.74%	0.3	Watch
10	*fosfomycin*	J01XX01	28249.33	1.70%	0.29	Watch
11	*cefditoren*	J01DD16	27080.00	1.63%	0.28	Watch
12	*doxycycline*	J01AA02	23600.00	1.42%	0.24	Access
13	*prulifloxacin*	J01MA17	22478.00	1.35%	0.23	Watch
14	*cefalexina*	J01DB01	12786.00	0.77%	0.13	Access
15	*ceftibuten*	J01DD14	11597.40	0.70%	0.12	Watch
16	*cefuroxima*	J01DC02	7326.00	0.44%	0.08	Watch
17	*cefpodoxime*	J01DD13	5865.00	0.35%	0.06	Watch
18	*minocycline*	J01AA08	5000.00	0.30%	0.05	Watch
19	*norfloxacin*	J01MA06	4256.00	0.26%	0.04	Watch
20	*moxifloxacin*	J01MA14	3925.00	0.24%	0.04	Watch
21	*spiramycin*	J01FA02	3040.00	0.18%	0.03	Watch
22	*lomefloxacin*	J01MA07	3005.00	0.18%	0.03	Watch
23	*roxithromycin*	J01FA06	2400.00	0.14%	0.02	Watch
24	*bacampicillin*	J01CA06	1968.00	0.12%	0.02	Access
25	*lymecycline*	J01AA04	1778.00	0.11%	0.02	Watch

26	*amikacin*	J01GB06	1162.50	0.07%	0.01	Access
27	*cefaclor*	J01DC04	1132.50	0.07%	0.01	Watch
28	*ceftazidime*	J01DD02	851.25	0.05%	0.01	Watch
29	*pipemidic acid*	J01MB04	610.00	0.04%	0.01	Watch
30	*miocamycin*	J01FA11	588.00	0.04%	0.01	Watch
31	*teicoplanin*	J01XA02	550.50	0.03%	0.01	Watch
32	*cefodizime*	J01DD09	549.00	0.03%	0.01	Watch
33	*cefepime*	J01DE01	492.75	0.03%	0.01	Watch
34	*clindamycin*	J01FF01	335.33	0.02%	<0.01	Access
35	*piperacillin+tazobactam*	J01CR05	328.00	0.02%	<0.01	Watch
36	*flucloxacillin*	J01CF05	258.00	0.02%	<0.01	Access
37	*cefotaxime*	J01DD01	207.50	0.01%	<0.01	Watch
38	*lincomycin*	J01FF02	206.00	0.01%	<0.01	Watch
39	*netilmicin*	J01GB07	167.43	0.01%	<0.01	Watch
40	*pefloxacin*	J01MA03	167.00	0.01%	<0.01	Watch
41	*rufloxacin*	J01MA10	120.00	0.01%	<0.01	Watch
42	*tobramycin*	J01GB01	107.71	0.01%	<0.01	Watch
43	*cefprozil*	J01DC10	81.00	<0.01%	<0.01	Watch
44	*josamycin*	J01FA07	72.00	<0.01%	<0.01	Watch
45	*telithromycin*	J01FA15	55.00	<0.01%	<0.01	Watch
46	*cefazolin*	J01DB04	35.67	<0.01%	<0.01	Access
47	*benzathine benzylpenicillin*	J01CE08	22.75	<0.01%	<0.01	Access
48	*oxacillin*	J01CF04	16.50	<0.01%	<0.01	Access
49	*erythromycin*	J01FA01	15.00	<0.01%	<0.01	Watch
50	*piperacillin*	J01CA12	14.43	<0.01%	<0.01	Watch
51	*ampicillin+sulbactam*	J01CR01	10.51	<0.01%	<0.01	Access
52	*metronidazole*	J01XD01	4.00	<0.01%	<0.01	Access
53	*cefoxitin*	J01DC01	2.50	<0.01%	<0.01	Watch

(c) Females

Rank	Antibiotic name	ATC 5th level	DDD		DID	AWaRe Class
			N	%		
1	*amoxicillin+clavulanic acid*	J01CR02	607175.99	30.24%	4.88	Access
2	*levofloxacin*	J01MA12	285625.00	14.22%	2.3	Watch
3	*clarithromicyn*	J01FA09	200494.00	9.98%	1.61	Watch
4	*azithromicyn*	J01FA10	181193.00	9.02%	1.46	Watch
5	*ciprofloxacin*	J01MA02	142540.00	7.10%	1.15	Watch
6	*amoxicillin*	J01CA04	135937.61	6.77%	1.09	Access
7	*cefixime*	J01DD08	132065.00	6.58%	1.06	Watch
8	*fosfomycin*	J01XX01	101353.33	5.05%	0.81	Watch
9	*ceftriaxone*	J01DD04	35185.50	1.75%	0.28	Watch
10	*cefditoren*	J01DD16	30380.00	1.51%	0.24	Watch
11	*trimetoprim+sulfamethoxazole*	J01EE01	29479.00	1.47%	0.24	Access
12	*doxycycline*	J01AA02	19280.00	0.96%	0.15	Access

13	*prulifloxacin*	J01MA17	18680.00	0.93%	0.15	Watch
14	*norfloxacin*	J01MA06	13811.00	0.69%	0.11	Watch
15	*ceftibuten*	J01DD14	13393.80	0.67%	0.11	Watch
16	*cefalexina*	J01DB01	10600.50	0.53%	0.09	Access
17	*cefpodoxime*	J01DD13	9004.00	0.45%	0.07	Watch
18	*cefuroxima*	J01DC02	8928.00	0.44%	0.07	Watch
19	*spiramycin*	J01FA02	4664.00	0.23%	0.04	Watch
20	*moxifloxacin*	J01MA14	3885.00	0.19%	0.03	Watch
21	*roxithromycin*	J01FA06	3798.00	0.19%	0.03	Watch
22	*minocycline*	J01AA08	2508.00	0.12%	0.02	Watch
23	*pipemidic acid*	J01MB04	2480.00	0.12%	0.02	Watch
24	*lomefloxacin*	J01MA07	2410.00	0.12%	0.02	Watch
25	*bacampicillin*	J01CA06	2364.00	0.12%	0.02	Access
26	*cefaclor*	J01DC04	1882.00	0.09%	0.02	Watch
27	*miocamycin*	J01FA11	1260.00	0.06%	0.01	Watch
28	*amikacin*	J01GB06	1043.50	0.05%	0.01	Access
29	*ceftazidime*	J01DD02	998.13	0.05%	0.01	Watch
30	*lymecycline*	J01AA04	910.00	0.05%	0.01	Watch
31	*cefodizime*	J01DD09	647.50	0.03%	0.01	Watch
32	*cefepime*	J01DE01	534.75	0.03%	<0.01	Watch
33	*cefotaxime*	J01DD01	500.50	0.02%	<0.01	Watch
34	*clindamycin*	J01FF01	459.00	0.02%	<0.01	Access
35	*piperacillin+tazobactam*	J01CR05	442.00	0.02%	<0.01	Watch
36	*pefloxacin*	J01MA03	405.00	0.02%	<0.01	Watch
37	*flucloxacillin*	J01CF05	360.00	0.02%	<0.01	Access
38	*lincomycin*	J01FF02	349.33	0.02%	<0.01	Watch
39	*teicoplanin*	J01XA02	289.00	0.01%	<0.01	Watch
40	*cefprozil*	J01DC10	204.00	0.01%	<0.01	Watch
41	*tobramycin*	J01GB01	140.00	0.01%	<0.01	Watch
42	*josamycin*	J01FA07	99.00	<0.01%	<0.01	Watch
43	*netilmicin*	J01GB07	91.86	<0.01%	<0.01	Watch
44	*cefazolin*	J01DB04	62.33	<0.01%	<0.01	Access
45	*piperacillin*	J01CA12	38.57	<0.01%	<0.01	Watch
46	*erythromycin*	J01FA01	27.00	<0.01%	<0.01	Watch
47	*telithromycin*	J01FA15	15.00	<0.01%	<0.01	Watch
48	*metronidazole*	J01XD01	15.00	<0.01%	<0.01	Access
49	*benzathine benzylpenicillin*	J01CE08	13.50	<0.01%	<0.01	Access
50	*rufloxacin*	J01MA10	12.00	<0.01%	<0.01	Watch
51	*ampicillin+sulbactam*	J01CR01	6.66	<0.01%	<0.01	Access
52	*cefoxitin*	J01DC01	1.00	<0.01%	<0.01	Watch
53	*oxacillin*	J01CF04	0.00	0.00%	0.00	Access
